# Co-administration of treatment for drug-resistant TB and hepatitis C

**DOI:** 10.5588/ijtld.22.0403

**Published:** 2023-01-01

**Authors:** I. D. Olaru, M. Beliz Meier, S. G. Schumacher, N. Prodanovic, P. J. Kitchen, F. Mirzayev, C. M. Denkinger

**Affiliations:** 1 Division of Infectious Disease and Tropical Medicine, Heidelberg University Hospital, Heidelberg, Germany; 2 Clinical Research Department, London School of Hygiene & Tropical Medicine, London, UK; 3 Global TB Programme, World Health Organization, Geneva, Switzerland; 4 German Center for Infection Research (DZIF), partner site Heidelberg University Hospital, Heidelberg, Germany

Dear Editor,

Of the 9.9 million individuals estimated to have developed TB in 2020, 3–4% had multidrug-resistant/rifampicin-resistant TB (MDR/RR-TB).^1^ In addition, hepatitis C virus (HCV) infection is not uncommon in patients with TB. Global HCV antibody seroprevalence among patients with TB was 10.4%, (personal communication, I.D. Olaru), which is higher than the prevalence of 1.4% in the general population.^2^ TB treatment can be problematic in patients with pre-existing liver disease due to the potential hepatotoxicity of drugs used for treatment and patients with HCV and TB are at increased risk for anti-TB drug-induced liver injury.^3^ The development of oral direct-acting antivirals (DAA) has revolutionised HCV treatment by improving treatment success and shortening treatment duration, with a much better safety profile and tolerability. Using DAAs, over 90% of patients achieve a sustained virologic response (SVR) i.e., clearance of the virus.^4^ In 2018, WHO screening and treatment guidelines recommend a universal treatment approach for all individuals with chronic HCV infection irrespective of disease stage (except young children and pregnant women).^5^ The expanded availability of generic DAAs and decrease in treatment costs for HCV treatment have improved access and led to an increase in people receiving treatment.^6^ We therefore conducted a systematic review to determine if co-administration of treatment for MDR/RR-TB and HCV can improve MDR/RR-TB treatment outcomes in co-infected patients. We also examined available data on the timing for initiating antivirals for HCV in relation to treatment for MDR/RR-TB.

Our review included studies conducted among patients with MDR/RR-TB and HCV infection who received concomitant treatment. Only treatment with DAAs was considered. Databases searched included Medline, EMBASE, Web of Science, Cochrane Library, African Journals Online, LILACS,WHO International Clinical Trials Registry, Clinicaltrials.gov and www.elibrary.rsl.ru (for articles in Russian) for articles published between 1 January 2014 (when DAAs were introduced in clinical practice) and 17 June 2021 (Supplementary Data Table S1). Following this, the search was updated to 15 March 2022. No language or geographical restrictions were used. Studies were eligible if they included patients with both MDR/RR-TB and HCV who received concomitant treatment with DAAs, provided MDR/RR-TB was microbiologically confirmed in most patients, and MDR/RR-TB treatment outcomes were reported according to HCV status. Studies without original data, or those with fewer than 10 patients, were excluded. The systematic review was registered on PROSPERO (CRD42021277283).

A total of 1,689 articles were identified during the database search, of which only two^7^,^8^ were retained for full text review (Supplementary Figure S1). By searching other sources, we identified an additional study (Supplementary Data Table S2).^9^,^10^ Only the latter study was eligible according to our selection criteria: other studies were ineligible because they included too few patients with known MDR-TB outcomes,^8^ or did not report outcomes.^7^ The study eligible for this review was conducted within a European collaborative network and included 23 patients from six centres in four countries (France, Italy, Belarus and Spain),^10^ of whom nine were also HIV co-infected, and one had hepatitis B and D infection in addition to HCV. All patients had pulmonary MDR-TB, with 4/23 also having extra-pulmonary involvement.

DAAs were initiated because of hepatotoxicity during MDR-TB treatment (*n =* 7) or because of elevated liver function test results prior to starting MDR-TB treatment (*n =* 6). Most patients received velpatasvir/sofosbuvir (*n =* 9) or daclatasvir/sofosbuvir (*n =* 8). At 4 weeks after DAA start, all patients had normalised liver function tests and undetectable HCV viral load at 12 weeks of treatment. HCV viral load at 12 and 24 weeks post-DAA completion were available for 11/23 patients and all had a sustained virological response. Bedaquiline was used for MDR-TB treatment in 15 patients, but it is unclear from the study report whether DAAs and bedaquiline were co-administered.

At the time of analysis, 11 patients had completed MDR-TB treatment and treatment was ongoing for the remainder. MDR-TB cure was achieved in 10/11 (91%) patients, with one additional patient dying of accidental causes. Liver-related adverse events were recorded in 11/23 patients, with most events being mild and occurring before DAAs were initiated. The authors concluded that co-administration of DAAs and MDR-TB treatment is safe and effective. The study by Melikyan et al.^7^ was excluded because it did not report MDR-TB treatment outcomes, but it has some important insights: during the study period (between 2016 and 2018), 30 patients received concomitant treatment for HCV and MDR-TB. Treatment for HCV was started within a median of 5.4 months (interquartile range 2.1–12.1) of MDR-TB treatment initiation. Patients received daclatasvir/sofosbuvir or ledipasvir/sofosbuvir. In patients with HCV genotype 3a infection and advanced fibrosis, ribavirin was also used. When considering only those with a measured viral load at 12 weeks, the SVR was 23/24 (95.8%). Of the six patients who were not tested at 12 weeks, four had a negative viral load at the end of DAA treatment. One patient experienced a serious adverse event (severe allergic reaction), possibly related to DAAs, which required interruption of DAAs and MDR-TB treatment. An additional four adverse events related to the MDR/RR-TB drugs were reported. Musso et al.^8^ report on two patients who received concomitant MDR-TB and HCV treatment, and are included in the study by Tunesi et al. described above.^10^

Our systematic review identified only one small study fulfilling the eligibility criteria. Two additional small studies described concomitant treatment and suggested that co-administration of treatment may be well tolerated and associated with SVR for HCV infection. Liver-related adverse effects were rarely reported. In the small number of patients with reported MDR-TB outcomes, treatment success was high. However, treatment was conducted in highly-specialised institutions with experience in the management of MDR-TB, which may have resulted in better treatment outcomes, therefore limiting general-isability. None of the studies compared outcomes of patients receiving co-administered treatment with those of patients receiving MDR/RR-TB treatment alone. While co-administration of rifampicin and HCV treatment is clearly contraindicated (due to the effect on cytochrome P450 (CYP) 3A4),^11^,^12^ few or no interactions are anticipated to occur with drugs for MDR/RR-TB treatment. Bedaquiline may have the potential for some interaction with DAAs (which are CYP enzyme inhibitors), but the effect is poorly understood.^12^ In the absence of high-quality evidence, clinicians from some settings initiate HCV treatment to mitigate hepatotoxicity and stop viral replication, probably reasoning that the overall benefits are likely to outweigh harms (Table). However, because of the general lack of data, increased pill burden and concerns around drug–drug interactions and treatment adherence, clinicians may hesitate to co-administer MDR/RR-TB and HCV treatment. Overall, concomitant treatment for HCV and MDR/RR-TB appears feasible, but the current evidence base is extremely limited. Furthermore, not all settings have DAAs available at affordable prices and treatment costs can vary widely, which may lead to challenges in accessing treatment for those who need it most.^6^

**Table:** Considerations on treatment co-administration for MDR/RR-TB and HCV.

Domain	Considerations
Treatment effectiveness	MDR/RR-TB treatment success: 59%^1^ (global average; success rates vary widely depending on regimen and patient and other contextual factors) SVR for HCV using DAAs >90% (may be lower for genotype 3)^4^
Adverse events	MDR/RR-TB treatment: hepatotoxicity variable depending on patient population and drugs used TB and HCV: 3.2 higher odds for DILI in TB patients with HCV than in TB patients without HCV^3^ MDR/RR-TB and HCV: DILI more common among MDR/RR-TB patients than among those without pre-existing liver disease^13^ MDR/RR-TB and HCV: liver function tests among MDR/RR-TB patients may normalise with DAAs^8^,^10^ Indirect evidence on HBV: TB and HBV: higher risk of severe DILI and liver failure in the absence of co-administration of treatment for HBV and TB;^14^ HBV treatment can halve the risk of hospitalisation due to liver injury^15^
Drug-drug interactions	TB and HCV: DAAs contraindicated (interaction with rifampicin)^11^ MDR/RR-TB: presumably safe; bedaquiline may interact with DAAs^12^
Adherence	↑ pill burden for co-administration Some DAAs available as fixed-dose combinations Patients at risk for HCV may be at ↑ risk of non-adherence Reducing the risk of hepatotoxicity-related side-effects may improve adherence to MDR/RR-TB treatment
Costs	↑ cost of drugs DAAs available as low-cost generics in LMIC

MDR = multidrug-resistant; RR = rifampicin-resistant; HCV = hepatitis C virus; SVR = sustained virologic response; DAA = direct-acting antivirals; DILI = drug-induced liver injury; LMIC = low- and middle-income countries.

Given the current state of evidence, no conclusion can be drawn with regards to co-administration of MDR/RR-TB and HCV treatment and MDR/RR-TB outcomes. This supports the need for future studies investigating the effect of co-administered treatment and the optimal timing for initiating DAAs in relation to MDR/RR-TB treatment start. This could be done by conducting larger observational studies exploring outcomes, safety, and optimal starting timepoints of co-administered treatment in different settings and patient populations. Considering the relatively high prevalence of HCV infection among specific subgroups of patients with MDR/RR-TB, our findings indicate that these patients may be receiving suboptimal care and demonstrate the need for more research to inform patient management.
